# Comparison of hyaluronic acid-containing topical eye drops with carbomer-based topical ocular gel as a tear replacement in canine keratoconjunctivitis sicca: A prospective study in twenty five dogs

**Published:** 2012

**Authors:** David Williams, Sheldon Middleton, Hamidreza Fattahian, Roozbeh Moridpour

**Affiliations:** 1 *Department of Veterinary Medicine, University of Cambridge, Madingley Road, Cambridge, England;*; 2 *Department of Surgery, Faculty of Specialized Veterinary Sciences, Science and Research Branch, Islamic Azad University, Tehran, Iran.*

**Keywords:** Keratoconjunctivitis sicca, Tear replacement, Ocular surface, Eye, Dog

## Abstract

The aim of this study was to evaluate the efficacy of a hyaluronic acid containing eye drop in ameliorating ocular surface pathology and discomfort in canine keratoconjunctivitis sicca (KCS). Twenty five dogs with KCS treated with a topical carbomer (CA)-based tear replacement gel were moved to treatment with a hyaluronic acid (HA)-containing tear replacement eye drop. Dogs were subject to a full ophthalmic examination at the beginning of the study and after two and four weeks of treatment, Schirmer tear tests (STTs) were performed at each examination. Conjunctival hyperemia, ocular discharge and ocular irritation were evaluated and scored on a 0-3 semi-quantitative scale. Values were compared before and after 4 weeks of treatment using a paired *t*-test. Evaluation scores were compared using the Wilcoxon signed rank test. The transfer from CA-based to HA-containing tear replacement significantly decreased the conjunctival hyperemia score from 2.12 ± 0.73 to 1.26 ± 0.59 and ocular discomfort was lowered from 2.11 ± 0.97 to 0.93 ± 0.75. Ocular discharge was reduced from a score of 1.04 ± 0.82 to 0.70 ± 0.53, however, the decrease did not reach statistical significance. Schirmer tear test was increased with statistical significance (*p* < 0.001) but given that the increase was only from 5.42 ± 3.50 to 6.19 ± 3.86 mm min^-1^; this was not considered clinically significant. This study demonstrated that HA-containing eye drops used twice daily in dogs with KCS had greater ameliorative effects on ocular surface health and discomfort than did CA-based topical gels used as or more frequently.

## Introduction

A significant number of dogs suffer from aqueous tear deficiency (keratoconjunctivitis sicca; KCS), with 4% of animals in a recent study demonstrating less than the optimal 15mm of tear wetting in a minute using the standard Schirmer tear test. The standard treatment for this condition is topical 0.2% cyclosporine in an ointment base (Optimmune, Intervet Schering Plough, UK) and in general this medication is highly effective in restoring normal or near-normal tear production and ocular surface health.^1^ However, between 5 and 10% of dogs affected by KCS referred to our clinic over the past five years do not respond adequately to this treatment and the ointment is expensive to use as a life-long therapy. Before the advent of topical cyclosporine, tear replacement was the only non-surgical therapeutic option and in cases where topical cyclosporine fails, it is the remaining medical treatment option. However, carboxymethyl cellulose and poly vinyl alcohol-based tear replacements available need to be given very frequently and are less than perfect substitutes for natural tears. Carbomer-based tear replacements are significantly more long-lasting but a number of owners find this gel-based therapy difficult to apply and many dogs prefer an eye drop formulation. Eye drops containing hyaluronic acid have proved to be efficacious in human patients and may be more beneficial in dogs with KCS than currently available topically medications. Here we sought to compare the treatment success of Clinadry, an HA-containing preservation-free eye drop with Viscotears, a carbomer-based tear replacement gel. 

In the United Kingdom and indeed throughout Europe, the ‘cascade’ system requires that conditions in animals are treated primarily with therapeutic agents which are licensed for veterinary use. This seeks to ensure that animals are treated with drugs for which efficacy and safety have been assured. Thus any new case of KCS must first be treated with 0.2% cyclosporine ointment (Optimmune, Intervet: Schering Plough, UK), no other agents being licensed in the UK for treatment of the condition. Only in cases where this treatment does not produce sufficient amelioration of ocular signs can another medication be used. Most animals in this situation have already been medicated with a topical carbomer gel, and the dogs used in this study were already being treated with Viscotears (Allergan Australia Pty Ltd., Gordon, New South Wales) or Lubrithal (Schering-Plough, Kenilworth, NJ, USA), the two most widely used carbomer gels in the UK.

We thus employed a cross-over strategy in which KCS-affected animals being treated with a carbomer gel twice or three times daily were transferred to treatment twice daily with the HA-containing tear replacement Clinadry (Ecuphar, Oostkamp, Belgium). Our aim was that after four weeks on this treatment, animals would be transferred back to their original treatment to assess differences in ocular parameters detailed below on both treatment regimes. However, only two of the twenty five owners were prepared to return to the carbomer-based medication, so pleased were they with the improvements in their animals ocular irritation when on the HA-containing drop formulation.

The single transfer experimental design used here clearly has disadvantages as does the non-masked nature of this study. Given the marked difference in the drop formulation of the HA-containing medication and the gel-based carbomer, a fully masked study was impossible, This clearly has an influence on both owner and veterinary observer’s assessment of ocular parameters, but given the highly significant improvement in conjunctival hyperemia and ocular comfort in this study, it is hoped that this study at least provides a preliminary basis for further better-masked evaluation of these novel tear replacement formulations.

## Materials and Methods

Twenty seven dogs were included in the study with 25 fulfilling more than one month on the treatment regime and 35 eyes meeting the criteria for involvement in the study, a Schirmer tear test of 10 mm min^-1^ or less and ocular surface pathology as detailed in table 1. Two dogs left the study one because of geographical relocation and one because of owner non-compliance with the dosing and re-examination regime.

Dogs were subjected to a full ophthalmic examination using direct and indirect ophthalmoscopy, slit lamp biomicroscopy, a Schirmer I tear test and fluorescein dye staining of the ocular surface to determine possible corneal ulceration. Eyes were scored by the examining veterinarian (DW) for degree of conjunctival hyperemia (CH), ocular irritation (OI) and ocular discharge (OD) on a simple scoring system of 0 (not present), 1 (mild), 2 (moderate) or 3 (severe). Irritation was assessed by documenting width of palpebral aperture, amount of blepharospasm and degree of self-trauma through rubbing of the ocular surface. Having been instructed on assessment of irritation at this first examination, owners were asked to keep a diary noting degree of ocular irritation and were asked at examinations after 2 and 4 weeks whether they perceived the ocular comfort was improved, the same or deteriorated compared with the previous status on the CA treatment, here with a score of 2 (much improved), 1 (improved), 0 (not changed), -1 (deteriorated) or -2 (markedly deteriorated).

Schirmer tear test (STT) values before and after HA treatment were compared using a paired *t*-test while given that data from the two eyes of the same dog are likely to be correlated, one KCS-affected eye was chosen randomly from each dog using a random number table to ensure that inadvertently low *p*-values would be calculated.^2^ The level of significance was set at *p *< 0.05.

## Results

The 25 dogs involved in this study were of a number of breeds, genders and ages as detailed in table 1 with an average age of 6.2 years. The average Schirmer I tear tests before and after HA treatment in the KCS-affected eyes are given in table 2 as are average scores before and after 4 weeks of HA treatment for CH, OI and OD.

**Table 1:** Details of dogs in study

**Breed**	**Gender**	**Age (year)**
**Lhasa apso**	Fn	8
**Yorkie**	Mn	12
**English Cocker Spaniel**	Fn	8
**labrador cross**	Fn	7
**Yorkshire terrier**	Me	11
**Boxer**	Me	6
**St Poodle**	Mn	4
**Cavalier king Charles spaniel**	Fn	2
**Pug**	Mn	10
**Corgi cross**	Mn	9
**English Cocker Spaniel**	Mn	2
**Shar Pei**	Fn	1
**French Bulldog**	Fn	6
**West Highland White Terrier**	Fn	8
**Shih Tzu**	Fn	8
**Labrador**	Mn	1
**German Shepherd Dog**	Fe	6
**Pug**	Fn	1
**Jack Russell Terrier**	Mn	8
**Jack Russell Terrier**	Mn	7
**Staffpordshire Bull Terrier**	Fn	9
**Rhodesian Ridgeback**	Fn	6
**Englsih Springer Spaniel**	Fn	7
**Bulldog**	Fn	2
**French Bulldog**	Fn	4

Fn= Female neutered

Fe= Female entire

Mn=Male neutered

Me=Male entire

The increase in STT values after 4 weeks of HA treatment was statistically significant at *p* < 0.001 as over half of the eyes experienced an increase in STT value although not clinically significant, given that the average value increased from 5.40 ± 3.50 to 6.20 ± 3.90 mm min^-1^. Conjunctival hyperemia and ocular irritation were significantly reduced as shown by the p values in Table 2. 

**Table 2:** Schirmer tear tests and ocular scores before and after HA treatment

**Parameters**	**STT (mm min** ^-1^ **)**	**Conjunctival hyperemia**	**Ocular irritation**	**Ocular discharge**
**Value before HA treatment**	5.42 ± 3.50	2.12 ± 0.73	2.11 ± 0.97	1.04 ± 0.82
**Value after HA treatment**	6.19 ± 3.86	1.26 ± 0.59	0.93 ± 0.75	0.70 ± 0.53
**Statistical significance**	P = 0.005	P = 0.00008	P = 0.0003	P = 0.11

## Discussion

The tear film used to be considered as a three layer covering to the cornea with a lipid layer externally, an aqueous layer providing the majority of the tear film thickness and then a mucin layer next to corneal epithelium itself. We now know from laser interferometry studies of the tear film that such a demarcated model is less than accurate.^3^ In fact the mucins in the tear film include species which adhere to the corneal epithelial surface and also those free in the aqueous layer. They are high molecular weight glyoproteins with between 20 and 200 amino acid repeats. So ideally a tear replacement should be a high molecular weight molecule with the ability to float free in the remaining aqueous tear layer present in a patient with dry eye but also to bind to the corneal epithelial cell surface.

Two candidates for such a replacement exist, one a carbomer gel in such formulations as Viscotears and Lubrithal and the second a formulation containing hyaluronic acid, here used as Clinadry. The carbomer in Viscotears or Lubrithal is a lengthy polyacrylic acid produced artificially. Hyaluronic acid, however, as employed in Clinadry, is generated using bacterial fermentation^4^ and as such retains the natural biological functions which allow it to bind more closely with the cell surfaces of the corneal surface. It is for this reason that hyaluronate eye drops have been shown to be somewhat superior to those containing carbomer molecules.^5^ They have a high residence time on the cornea allowing for less frequent application than other tear replacing formulations and also in human studies give less of a blurring action on vision, suggesting a closer appropriation to the corneal surface.^6^ A study examining the spreading of tear replacements across the ocular surface demonstrated that while aqueous tear replacements spread unevenly across the corneal surface, leading to blurring and lower visual acuity, sodium hyaluronate containing tear replacements spread more evenly over the corneal surface, ensuring that all areas of the ocular surface were equally protected by the applied drop.^7^

The long chain length of these hyaluronic acid molecules influences their spreading activity and their viscosity. They have what is known as non-Newtonian rheological properties; basically since the long molecules tangle with each other such fluids do not flow as readily as liquid containing smaller molecules. Thus as well as adhering to the corneal surface these molecules are retained better in the fluid phase of the tear film, just as natural mucins would be in the normal eye.

*In vitro* studies have shown that hyaluronate improves epithelial cell migration such as is required in corneal ulcer healing.^8^ Ultrastructural studies show fewer deleterious changes to epithelial cells of the corneal surface using sodium hyaluronic acid compared to other tear replacements, which is what one might expect given the greater protective effects of a molecule that stays more tightly bound to the ocular surface and has a longer half-life in the fluid covering the ocular surface.^9^

A significant problem with most topical ocular medications is the need to include preservative and stabilisers and in particular a compound known as benzalkonium chloride. This quaternary ammonium compound is basically a bacteriostatic detergent and thus it is not surprising that it has deleterious effects on the corneal surface and especially on migrating corneal epithelial cells.^10^ One recent study showed benzalkonium chloride to kill between 56 and 89% of epithelial cells in an *in vitro* culture system^11^ while a similar study comparing the cytotoxicity of topical ocular medications containing benzalkonium chloride and those which were preservative free showed significantly less deleterious effect with the latter products.^12^ No wonder ophthalmologists are asking more and more for preservative-free ophthalmic preparations. Just such a formulation is available in this hyaluronic acid tear replacement. 

One of the main side effects of benzalkonium chloride is that it can induce apoptosis, pre-programmed cell suicide, in migrating epithelial cells.^13^ Such cell death is exactly what must be avoided in a situation such as dry eye where cell survival is compromised by the exposure resulting from tear deficiency. Sodium hyaluronate has the opposite effect inhibiting corneal epithelial cell apoptosis.^14^

Thus from many perspectives; from that of low frequency of application to the lack of detrimental effects on the corneal epithelium a tear replacement containing sodium hyaluronate in a preservative-free formulation offers an ideal tear replacement for the many dogs affected by keratoconjunctivitis sicca.
